# Accurate Quantification of Functional Analogy among Close Homologs

**DOI:** 10.1371/journal.pcbi.1001074

**Published:** 2011-02-03

**Authors:** Maria D. Chikina, Olga G. Troyanskaya

**Affiliations:** 1 Department of Molecular Biology, Princeton University, Princeton, New Jersey, United States of America; 2 Lewis-Sigler Institute for Integrative Genomics, Princeton University, Princeton, New Jersey, United States of America; 3 Department of Computer Science, Princeton University, Princeton, New Jersey, United States of America; New York University, United States of America

## Abstract

Correctly evaluating functional similarities among homologous proteins is necessary for accurate transfer of experimental knowledge from one organism to another, and is of particular importance for the development of animal models of human disease. While the fact that sequence similarity implies functional similarity is a fundamental paradigm of molecular biology, sequence comparison does not directly assess the extent to which two proteins participate in the same biological processes, and has limited utility for analyzing families with several parologous members. Nevertheless, we show that it is possible to provide a cross-organism functional similarity measure in an unbiased way through the exclusive use of high-throughput gene-expression data. Our methodology is based on probabilistic cross-species mapping of functionally analogous proteins based on Bayesian integrative analysis of gene expression compendia. We demonstrate that even among closely related genes, our method is able to predict functionally analogous homolog pairs better than relying on sequence comparison alone. We also demonstrate that the landscape of functional similarity is often complex and that definitive “functional orthologs” do not always exist. Even in these cases, our method and the online interface we provide are designed to allow detailed exploration of sources of inferred functional similarity that can be evaluated by the user.

## Author Summary

Common ancestry is a central tenet of modern biology, as genes from different species often show a high degree of sequence similarity, making it possible to study analogous processes across model organisms. However, many genes belong to large families with several duplicates and the relationship between genes from different species is often not one-to-one, complicating the transfer of experimental knowledge. We present a method that uses a large compendia of high-throughput expression data, that covers many genes that have not been analyzed in any other way, to systematically predict which genes are most likely to participate in the same biological process and thus have analogous function in different organisms. We show that our method agrees well with current experimental knowledge and we use it to investigate several families of genes that demonstrate the complexity of functional analogy.

## Introduction

The idea that protein sequence similarity implies shared function is a central paradigm in modern biology, allowing experimental knowledge obtained from model organisms such as yeast or mouse to be applied to our understanding of human diseases, or to be transferred via functional annotations to newly sequenced genomes. When no clear one-to-one homology relationship exists, however, and proteins of interest belong to families with several paralogous members (which may have arisen from post-divergence duplications), our ability to correctly transfer functional annotations based on sequence-similarity is fundamentally limited.

Such difficulties regularly arise in model organism studies of disease, where it is essential to identify which proteins and pathways are functionally analogous to the mammalian protein of interest. Attempts to understand human laminopathies through studies in Drosophila, for example, are limited by the lack of knowledge of the relationships among the human and fly lamin families; no one-to-one orthology exists in this case, and the most promising approach seems to be one based on functional information, rather than sequence. Frequently, however, not enough directed experimental information is available to make an accurate comparison among all homologs, as it is often the case that some members of homologous families are much better studied than others (see Figure S1 for a quantitative assessment). Thus, the possibility of leveraging high-throughput information to improve functional coverage is of significant interest.

Prior efforts at identifying functional orthologs (i.e. proteins that not only share sequence ancestry but also perform the same function) have investigated the technical aspects of global network alignments, largely focusing on large-scale protein-protein (physical) interaction networks [1], [2], [3], [4]. While PPI network alignments have been shown to yield orthology assignments that better conserve protein function when compared to using sequence similarity alone, such approaches have several limitations. Though it is constantly improving, the coverage of PPI networks is currently quite biased. Close homologs often differ widely in the number of reported interactions (Figure S2), and some proteins must often be excluded from consideration altogether because their interactions have not been assayed. On the other hand, protein interactions are often assayed under non-native conditions, potentially leading to the measurement of interactions that never occur biologically in spite of being chemically possible. Thus crucial information regarding cellular context, such as tissue specificity, may be entirely ignored when making PPI based orthology assignments.

We address these issues by developing a new local approach to alignment that leverages large collections of diverse gene expression data to identify functionally analogous homologs whose relevance to a particular research context can be easily interpreted. Microarray data is a complementary source of high throughput functional information that is in many cases as accurate as large-scale protein-protein interactions for predicting function [5], [6] and presents several advantages over PPI networks. It is one of the most unbiased and complete sources of functional information, as microarray experiments typically cover a large fraction of the genes in the genome, providing functional information about genes that have not been studied in any other way. Microarray studies are preformed with genes acting in their native context, preserving information about cellular state, tissue and developmental stage.

Identifying functional orthologs based on integrative analysis of large microarray compendia presents new challenges, including dense, hard to align networks, need for robust integration that is comparable across organisms, and robust identification of similarly functioning proteins. While we focus our discussion on microarray data because it provides the most unbiased coverage, our Bayesian integration method can readily combine different data types. In fact, in our online interface we provide both microarray-only predictions and predictions that incorporate PPI information. Furthermore, while in this study we focus on microarray data because of its excellent coverage in model organisms, our approach can easily integrate other expression data such as RNA-seq as it becomes widely available [7].

We employ a local alignment to provide a robust measurement of functional similarity among homologous proteins. This is in contrast to earlier work [1], [3], [4], [8], [9] that focused on global network alignment. The richness of expression data make global alignment methods both impractical and ultimately undesirable, since expression similarity may not be easily reduced to one-to-one alignment (see relevant results section). We develop and provide evaluation methods and functional gold standards for benchmarking prediction of functionally analogous proteins. While we demonstrate that our network similarity (NS) score accurately recapitulates experimental observations, we view it as evidence of functional analogy (not of one-to-one correspondence) and provide an exploratory interface that allows biologists to trace the sources of inferred similarity and evaluate its relevance to their area of interest.

## Results

Microarray studies provide a global view of the transcription state and are informative of the biological processes required by the tissue, cell-type or a particular perturbation under study. For this reason, individual microarray experiments have been used extensively to predict gene function [10], [11], [12], [13] and study its evolution [14], [15], [16]. However, direct comparison of gene-expression patterns across genomes is challenging, as experiments must be carefully selected so that conditions and treatments coincide, which may not be possible for certain organisms and is further complicated by different timing of response to perturbations. Moreover, many species-specific microarray experiments have no obvious analog in other species but may nevertheless provide useful information about gene function.

We provide a general approach that requires no direct alignment and seamlessly integrates the entirety of microarray data available for each species. This approach extends our previous work in large-scale microarray integrations to a multi-species comparison, allowing us to combine the breadth of microarray coverage with a network alignment approach to provide a global view of functional similarity across organisms.

**Figure 1:**
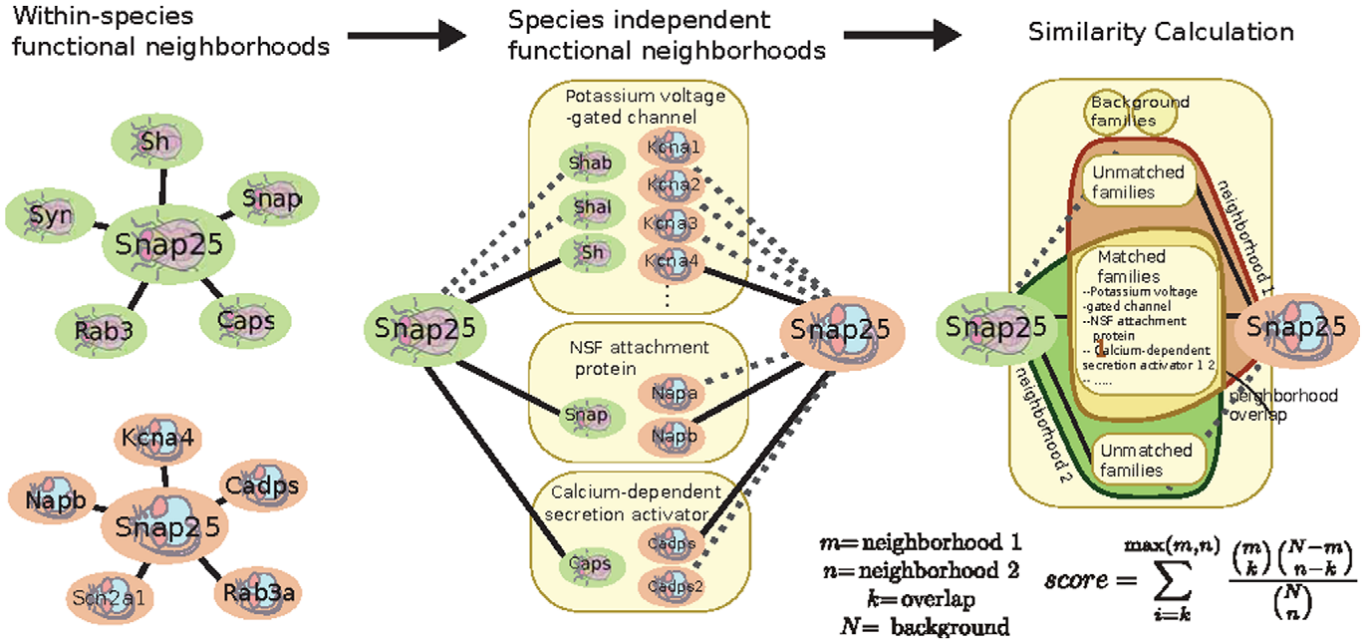
Overview of the functional similarity calculation method. Species-specific functional networks are derived by Bayesian integration of microarray data. For each intra-species pair of genes, the networks associate a probability of functional relationship based on their pattern of correlation. For a single gene, the set of genes with high probabilities of being functionally related to it defines a functional neighborhood. To make functional neighborhoods comparable across organisms, neighbors are grouped into meta-genes according to their Treefam families. The network similarity score is then defined as the hypergeometric probability of the overlap obtained from intersecting the sets of species-independent Treefam families present in each species-specific functional neighborhood. Such intersection analysis enables identification of specific biological processes responsible for network similarity scores. We have taken a comparison between the mouse and fly Snap25 genes as the basis for the schematic figure. The overlap meta-genes are a selection and the complete overlap can be viewed online using our webserver.

Our method (Figure 1) relies on comparisons of genes in co-expression space, which no longer requires that similar experiments are performed in all species, or for the experiments to be carefully aligned. For each organism we generate a probabilistic functional network by performing a Bayesian integration of a large compendium of microarray data. The Bayesian method interprets a correlation measure as evidence that the two genes are functionally related, *i.e.,* participate in the same biological process, possibly evidenced by genetic interaction or similar phenotype. However, though genes with similar functions often correlate, exactly how much evidence each correlation measure contributes depends on the quality/accuracy of each dataset, as determined by how well the individual dataset recapitulates known functional interactions (we use GO co-annotations, see Methods). The variability in the signal to noise ratio as well as the breadth of responses covered is statistically accounted for in such integrations of microarray data, which have been previously shown to provide accurate functional information that improves upon naïve correlation measures [17], [18]. The resulting functional network allows the function of a gene to be inferred from its neighborhood by the guilt-by-association principle. To allow the comparison of such signatures across species, we make use of a set of ‘meta-genes’, a multi-organism group of related genes, to provide organism independent gene equivalency classes, (defined as Treefam families [2]). Thus, each gene has an associated neighborhood (in our Bayesian integrated networks, this corresponds to a probability value cutoff) of same-species genes that is indicative of the gene's function, and the list of meta-genes represented in the neighborhood provides a species-independent functional signature. The functional similarity score between two genes is then defined as the hyper-geometric probability of the overlap of their neighborhoods' associated lists of metagenes. Intuitively speaking, we expect that two functionally similar genes in different organisms should have similar lists of meta-genes associated with their functional neighborhoods.

Figure 1 demonstrates a realistic scenario for neighborhood overlap computation. Here we consider a fly and mouse versions of the neuronal vesicle fusion gene, named Snap25 in both species. The two genes have neighborhoods consistent with their known functions which translate into similar meta-gene signatures. For instance, both genes are predicted to be functionally related to other members of the secretory machinery as well as general neuronal genes such as voltage gated ion channels. Indeed, the neighborhood overlap is much higher than expected by chance (p<10^-9^). However sequence analysis predicts that the Snap25 genes are not one-to-one orthologous as, in both organisms, they are part of a lineage specific duplication (with Snap23 in mouse and Snap24 in fly forming the rest of the co-orthologous group). Using the neighborhood overlap approach, we are able to confirm functional similarity between the two Snap25 genes as well as extend the analysis to all pairs of family members, revealing a surprising pattern of convergent evolution (see relevant section below for detailed discussion).

### Evaluation of performance of the network similarity score

While it has been shown that microarray data can been used to accurately distinguish functionally interacting gene pairs from unrelated ones, it is significantly more difficult to demonstrate that an integration successfully detects the subtle differences in the functional relationships between homologous proteins. Solving this evaluation problem is a prerequisite to providing functional analogy predictions that can be trusted in cases where a sequence comparison is ambiguous, as there is no reason to believe *a priori* that functional predictions which are accurate on average over all genes will be accurate on average over families of close homologs.

While sequence based analyses are indispensable for identification of molecular functions or domain architecture of proteins, organisms often possess several genes that belong to a cohesive family whose members are predicted to have the same structural or enzymatic features, which are nevertheless involved in quite different biological processes. Consider, for example, the mouse genes Snap25 (discussed briefly above) and its paralog Snap23. The two proteins have the same structural features [19], [20], interact with many of the same secretory proteins (BioGrid human and mouse) [21] and can complement one another in some assays [22]. However, physiologically they play quite different roles with Snap25 being involved in synaptic exocytosis while Snap23 is involved in a diverse array of trafficking processes in other tissues and cell types [23], [24], [25], [26], [27].

We hypothesize that our method, based on genomic datasets, is especially useful for the differentiation of such genes, as it provides a complementary characterization of function by specifically probing biological responses that may discriminate genes that otherwise appear similar at the sequence level. As this approach would be the most valuable for closely related genes (as distantly related genes can be distinguished based on sequence alone), which are very hard to distinguish functionally based on sequence, we focus our analysis on homologs that belong to the same TreeFam family.

Our first evaluation method is based on the tissue-expression pattern of genes. This evaluation is motivated by the fact that cross-species homologs that perform the same function are expected to express in similar tissues, reflecting the specific molecular requirements of different cell types. Correctly identifying homologous genes with similar tissue expression patterns is also a worthwhile goal in its own right, as tissue-specific expression is a critical facet of complex human disorders such as cancer and diabetes [28], [29].

**Figure 2:**
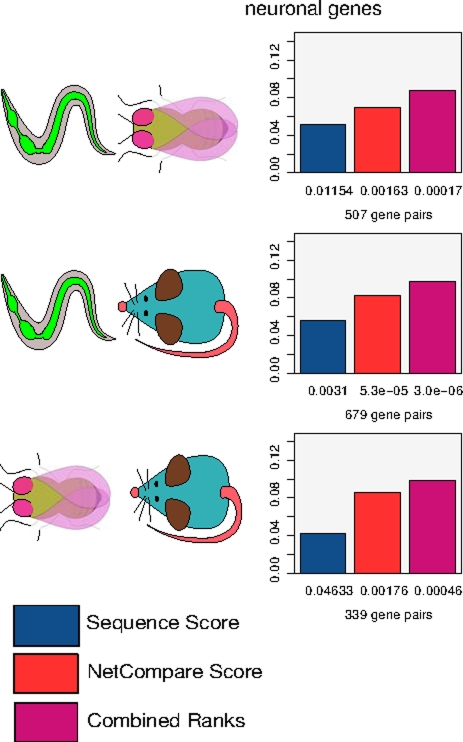
Network similarity score correctly identifies homologs with shared expression in the nervous system. We consider single query genes that are known to express in the nervous system and have multiple homologs in another organism (according to Treefam family co-membership), with at least one of the homologs also expressed in the nervous system (“correct” functional homolog), and another whose expression has been evaluated but was not detected in the nervous system (“incorrect” functional homolog in this evaluation). We then evaluate how well the various metrics rank the homologs consistent with their nervous system expression by computing the AUCs of homolog rankings (normalized per query gene). Numbers below the bars represent the p-value that corresponds to the AUC score.

Although the anatomies of worm, fly and mouse are quite different, and many tissues in one organism have no obvious analog in another, all three organisms possess a nervous system. We thus use fly and mouse genes annotated with brain expression and worm genes annotated with neuronal expression (all based on small-scale experiments such as in situ or GFP tagging) to define a nervous system standard. (Standard data is available in the download section of the website.) We then evaluate our predictions by assessing whether nervous system expressed genes are predicted by our method to have greater similarity to their homologs that are also present in the nervous system than to those which are not. Our method is significantly better than chance at matching pairs of nervous-system-expressed homologs. Moreover, when we subject a sequence derived measure to the same evaluation, we find that our network similarity score (based solely on the microarray-based network similarity) outperforms sequence in all comparisons (Figure 2).

It is important to note that our approach is complementary to—and can be used side by side with—sequence-alignment based methods. In fact, as our network similarity score and sequence-based scores provide orthogonal information, we find that a simple combined rank score can improve performance further in cases where both scores perform comparably-well (as evident in the fly/worm nervous system comparison, Figure 2).

**Figure 3:**
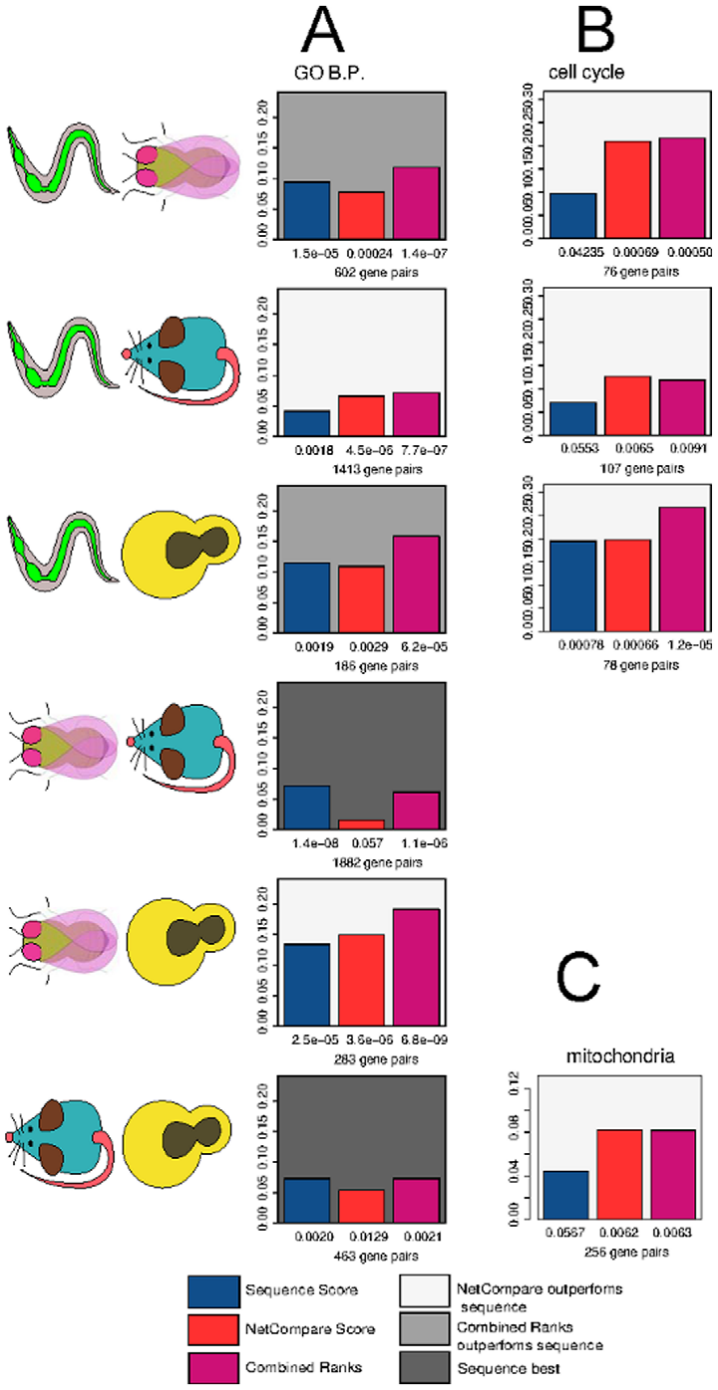
Network similarity score effectively identifies homologs involved in the same biological process and is often complementary to sequence-based information. This evaluation is performed identically to the nervous system evaluation with the variation that “correct” functional homologs are those that are co-annotated with the query to a specific GO term while “incorrect” ones are those that are annotated to a specific term but do not share annotations with the query. Numbers below the bars represent the p-value that corresponds to the AUC score. A. Evaluation performed with all of specific biological process annotations with experimental evidence codes. B. Evaluations performed by considering co-annotation to “cell cycle” only. C. Evaluations performed with co-annotation to “mitochondria”.

In addition to finding that our method can accurately pair homologs that are expressed in the same tissues, we aim to assess whether we are able to correctly identify cross-species homologs that play the same biological role in a cell. Thus, we also design an evaluation based on the Gene Ontology annotations, using a standard in which homologs that share “biologically specific” (i.e. sufficient for follow-up experiments, see Methods) GO Biological Process annotations are considered positives, while homologs that have been studied but do not have any specific annotations in common are considered negatives. This evaluation presents more challenges for our method since, in spite of the fact that we have excluded annotations based on sequence from the standard, it is likely that significant sequence bias remains, as sequence similarity often influences which proteins are studied experimentally and how they are annotated in the Gene Ontology. Nevertheless, our network similarity score still performs significantly better than background for nearly all comparisons when evaluated against this standard, and in many cases our network similarity score—or at least the combined network and sequence ranks score—outperforms sequence alone (Figure 3).

Detailed analysis reveals that there are areas of biological annotations where our network similarity score is especially accurate (as compared to sequence similarity) at identifying functionally analogous homologs. For example, if we use a standard based just on the “cell-cycle” GO annotation, the NS score performs well for all comparisons involving *C. elegans*. The GO evaluation standard for the cell-cycle process is likely unusually unbiased for *C. elegans*, as this process has been studied extensively in worm due to the ability to perform genome-wide RNAi screens [30]. This suggests that such pockets of strong performance could in fact be reflective of the real power of our method, which in other cases may be obscured by incomplete and biased evaluation standards.

To support this notion, we perform an evaluation using mitochondrial localization annotations, focusing on the homologs in mouse and yeast as these are the only two organisms in which genome-wide screens for mitochondrial localization and function have been performed [31], [32], [33], [34]. Despite the fact that the network similarity score does not appear to outperform sequence in the comparison of these two organisms in a GO-based evaluation, it is better than sequence at pairing homologs that both localize to the mitochondria, further lending evidence that the NS score performance is under-estimated by the GO standard due to annotation bias. Results for all evaluations performed in this study, including those for datasets generated using PPI information and additional process-specific standards, are presented in Table S1.

### Using the network similarity score to identify patterns of functional similarity not apparent from sequence-based comparisons: Convergent evolution in the Snap25 family

We have shown that the network similarity score provides reliable functional information that is complementary to sequence-based comparisons, correctly differentiating homologs with shared tissue-specific expression and playing similar roles in biological processes. We now illustrate how our method may be used to gain insight into the functional landscape of protein families with complex evolutionary histories.

**Figure 4:**
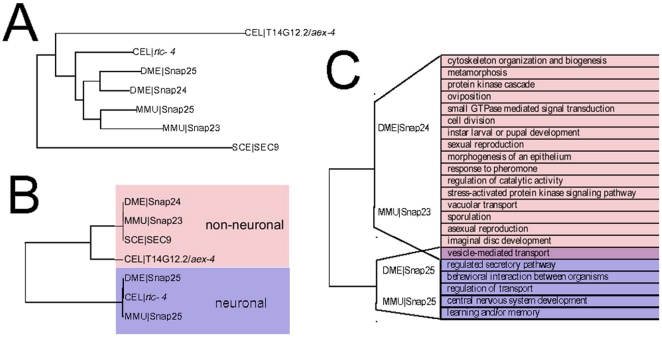
Convergent evolution in Snap25 family. A. The sequence derived family tree (TreeFam) indicates the presence of 2 lineage specific duplications so that the fly and mouse family members are collectively coorthologous. B. Using our method we have clustered members of the Snap25 family with respect to functional similarity. Family members cluster into neuronal and non-neuronal functional groups in a manner that is independent of their evolutionary history. Though the mouse and *Drosophila* Snap25 members have arisen independently by lineage specific duplications the expression of the two duplicates follow similar patterns with one homolog having neuronal pattern of expression, while the other expression pattern is consistent with participating in general exocytosis. C. Neighborhood GO enrichment for the four coorthologous genes. Functions shown are enriched in the neighborhood of either 2 or 4 (as in the case of “vesicle-mediated transport”) genes. While all four genes have neighborhoods indicative of secretary function the Snap23/Snap24 and Snap25/Snap25 pairs associate with a number of distinct functions. This analysis thus makes the prediction of convergent functions that is supported by several lines of experimental evidence [26], [27], [35], [38], [41].

We first consider the family represented by the mouse gene Snap25, a SNARE protein that participates in the regulation of synaptic vesicle exocytosis [35] and is the target of the Botulinum toxin A [36]. The inferred evolutionary history (Treefam) of the family is shown in Figure 4A. The functional similarity among these genes follows a surprisingly different pattern (Figure 4B): when the network similarity score is used to cluster the genes, two clear classes emerge. (Note: while our method is targeted towards evaluating homologs from two different species, we use our meta-gene approach to compute the neighborhood overlap score within-species to provide a family-wide clustering in visualizations.)

In particular, mouse Snap25 shows strong functional similarity to fly Snap25 but has no significant similarity to mouse Snap23 or fly Snap24, which are nevertheless similar to each other. This is particularly unexpected since multiple alignment analysis shows that the four genes arose from a single ancestor with subsequent lineage-specific duplications (Figure 4A). Despite the lack of direct evolutionary relationships defining the functional classes we have inferred, this division identified by our method is supported by experimental evidence. The mammalian and *Drosophila* Snap25 are predominantly neuronal, localize to the synapse, and mediate synaptic exocytosis [23]. However, both Snap23 and Snap24 have broader expression patterns and contribute in diverse processes. Snap23 is ubiquitously expressed [24] and has been shown to participate in glucose uptake in adipocytes [27] and platelet and mast-cell secretions [26], [37] and is localized to cell bodies in neurons [25]. Likewise *Drosophila* Snap24 appears in cell bodies of neurons and is expressed in other cell types, and has been shown to be involved in salivary gland exocytosis [38]. Thus the Snap25 cluster is comprised of neuron specific genes, while the Snap23/Snap24 cluster consists of genes that partake in regulated exocytosis in other cell types.

The distribution of yeast and *C. elegans* members among these functional classes is also consistent with this view. The yeast SEC9 gene shows strong functional similarity to non-neuronal family members, while the *C. elegans* family member *ric-4* that appears in the neuronal cluster is expressed exclusively in neurons [39]. The other *C. elegans* member (*aex-4*) clusters with non-neuronal genes, though it does show similarity with mouse Snap25 and *ric-4*. The tighter association with non-neuronal genes is supported by experiments that demonstrate that *aex-4* expresses in intestine, where it functions in signaling between the intestine and neurons that regulate defecation [40].

Since the fly and mouse homologs are predicted to have arisen by lineage-specific duplications, it appears that the independent emergence of neuronal and non-neuronal genes is an instance of convergent evolution. Interestingly, the development of both these types of Snap25 homologs may also be evident in the two *C. elegans* homologs, despite the fact the two genes appear to have arisen from a much earlier duplication. This observation raises intriguing questions regarding what biochemical constraints could be driving the emergence of the neuronal/non-neuronal specializations. It has recently been shown that the tissue-expression and functional role of the mammalian proteins parallels their calcium sensitivities [41], and it is possible that similar constrains hold in other organisms as well. In any case, we hope that our method will allow further examination of questions regarding convergent evolution of homologs, which by their nature cannot be addressed by sequence-based approaches alone.

### Using the functional similarity metric to find homologs of disease causing genes: A case study of the lamin family

Protein families with several lineage-specific duplications present a particular challenge for the transfer of disease models between organisms, since sequence similarity produces ambiguous mappings in such cases. For example, there has been significant interest in generating Drosophila models of vertebrate laminopathies that has been complicated by the lack of one-to-one orthologs. Vertebrate lamins have been classified into two types, type-A and type-B. Mutations in type-A cause a large class of diseases collectively termed laminopathies, such as muscular dystrophy and premature aging, while viable type-B mutations are extremely rare (the two B-type genes together are required for cellular viability while type-A lamin is not). Two main attributes are associated with the type-A/type-B distinction. First, B-type lamins are expressed ubiquitously while A-type lamins have a dynamic developmental expression profile. Second, type-B lamins possess a CaaX box that is prenylated and anchors the protein to the nuclear envelope, while mature type-A proteins do not [42].

Unlike many other invertebrates that have a single lamin gene, the *Drosophila* genome has two lamin genes (Lam and LamC) that resemble type-B and type-A lamins, respectively. Lam is ubiquitously expressed and has a CaaX box while LamC is developmentally regulated and lacks the anchor motif [43]. However, despite the striking similarities, the two types of lamins appear to have developed independently in the *Drosophila* and vertebrate genomes [2], leading to debate in the literature regarding how to best model vertebrate laminopathies in *Drosophila* [44], [45], [46].

**Figure 5:**
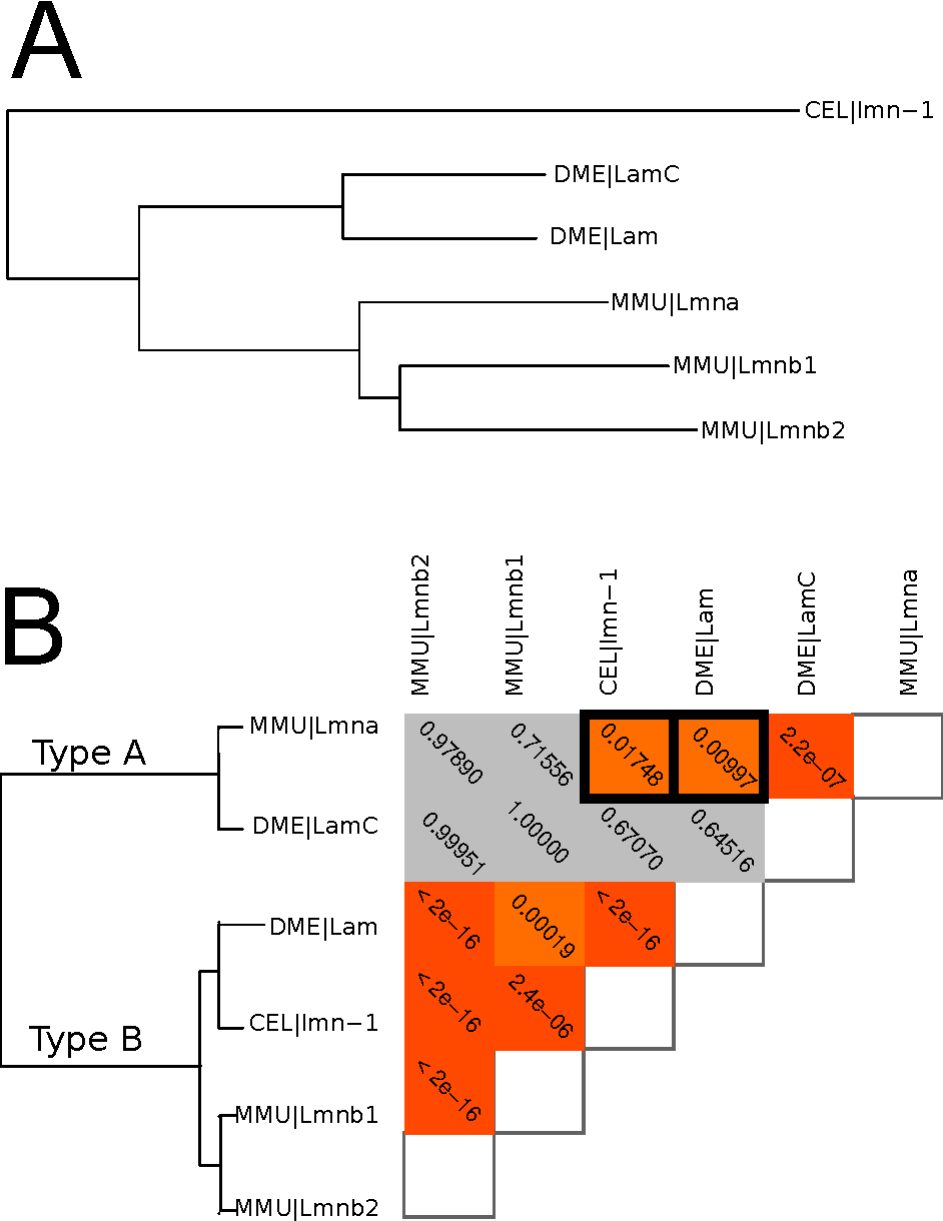
Functional similarity among members of the lamin family. A. The sequence derived family tree (Treefam) for the lamin genes being considered. B. The patterns of functional similarity among members of the lamin family. Lamins can be broadly classified as type-A and type-B based on pattern of expression and structural features, with type-A lamin mutations causing a diverse set of human diseases. While *C. elegans* has only a single type-B gene, the *Drosophila* genome has two lamin genes, Lam and LamC, that confirm to type-A and type-B patterns respectively, though they arose independently from their vertebrate counterparts. Using the network similarity score, we demonstrate that canonical invertebrate type-B lamins show significant similarity with mammalian type-A lamins and thus may be important in modeling human laminopathies.

Using our NS score to cluster mouse, *Drosophila,* and *C. elegans* lamins we are able to recapitulate the global type-A/type-B pattern: mouse Lmna clusters with *Drosophila* LamC, while mouse Lmnb genes cluster with *Drosophila* Lam and *C. elegans lmn-1* (which is also ubiquitously expressed and possesses a CaaX box, and thus is classified as Type-B [47]). However, our analysis also shows a surprising level of similarity between Mouse Lmna and the invertebrate type-B lamins, suggesting a more complex pattern of functional similarity (Figure 5).

The similarity between Lmna and *lmn-1* is perhaps not surprising, since *lmn-*1 is the only *C. elegans* lamin and thus must perform both type-B and type-A functions. The similarity between Lmna and Lam is more intriguing, however, as it suggests that no single functional homolog of vertebrate type-A lamin exists in the *Drosophila* genome. This is supported by the diverse phenotypes produced by Lam and LamC mutations in *Drosophila.* Mutations in LamC cause muscle nuclear envelope defects similar to those of the vertebrate A-type lamins [44], while Lam mutations cause locomotion defects and premature aging [45]. Consistent with these observations, our analysis suggests that while LamC is purely a type-A lamin, some type-A functions may be performed (possibly exclusively) by Lam alongside its role as a Type-B lamin, suggesting it could play an important role in understanding human type-A laminopathies.

### Evaluating the sources of network similarity scores: Case study of cytosolic superoxide dimutases

While we have shown that the network-based score can aid in finding homologs that perform the same function and have similar phenotypes, the nature of functional conservation is complex and may not be easily summarized with a single score, as demonstrated by the lamin example. Our method was in fact designed with this challenge in mind. Once genes from different organisms are made comparable by projecting their correlation neighborhoods onto organism-independent meta-genes, our network similarity score is generated using the neighborhood overlap metric (see Methods) that allows one to investigate which specific genes contribute to the functional similarity between two homologs. Such investigation is not easily possible if we apply other similarity metrics to the meta-gene neighborhoods, such as correlation (whose use would otherwise have little impact in the performance of our method in the evaluations described above). Our choice of the neighborhood overlap metric is primarily to allow detailed investigations of the underlying “reasons” for high network similarity scores.

To enable biologists to easily perform such analysis, we have made our method accessible through an interactive web interface that not only provides network similarity scores, but also allows the user to explore the source of inferred similarities. The user can identify precisely which meta-genes connections are shared by a pair of homologous genes and evaluate whether the overlap is representative of the particular biological functions that the user is interested in.

**Figure 6:**
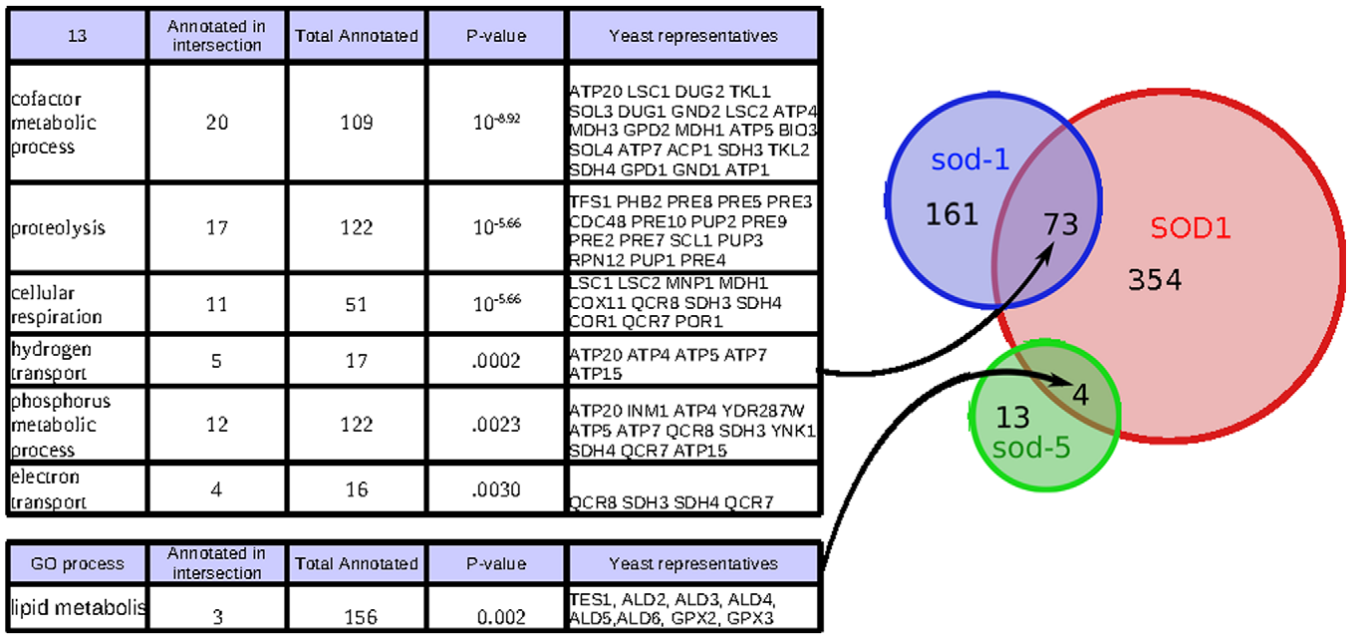
Using neighborhood overlaps between members of the cytosolic superoxide dismutase family to identify sources of functional ortholog similarity. A Venn diagram of shared meta-gene neighbors is shown. While both *C. elegans* genes have neighborhoods that overlap with SOD1, the overlap regions are distinct and have different functional enrichments that are consistent with the specialized functions of these genes.

As an example we consider the gene SOD1 in *S. cerevisiae*, which is the only representative of the cytosolic superoxide dismutase family. In contrast to *S. cerevisiae* and other organisms, *C. elegans* possesses two genes that belong to this family, *sod-1* and *sod-5*. The web interface allows one to explore the functional relationship among these three genes. As shown in Figure 6, *sod-1* and *sod-5* overlap completely disjoint functional regions of the SOD1 neighborhood in a manner consistent with what is known about their function. *sod-1* functions during reproductive growth, when the animal is most metabolically active, while *sod-5* is most active in the diapausal dauer stage [48], an alternative larval stage induced by starvation, when the animal does not feed and must rely on internal lipid stores. Consistent with these functional differences, the intersection of the neighborhoods of yeast SOD1 and worm *sod-1* is enriched for terms associated with high metabolic activity and contains clusters of genes that encode for enzymes in the TCA cycle, such as malate and citrate dehydrogenases.

In contrast, the overlap between yeast SOD1 and worm *sod-5* contains meta-genes that have been implicated in lipid metabolism, such as the yeast TES1 (a peroxisomal acyl-CoA thioesterase), members of a glutathione peroxidase family (TF105318) and aldehyde dehydrogenase family (TF300455). In spite of the fact that *sod-1* has a very large and significant intersection with SOD1, none of these genes associated with lipid metabolism are among the top interactors of *sod-1* (F54D8.3, an aldehyde dehydrogenase, ranks 1256^th^ while all others rank bellow the top 5000). Since lipids are not a major source of energy storage for *S. cerevisiae*, lipid metabolism and TCA cycle are typically active concurrently and are not separated in our compendium of expression data. In *C. elegans*, however, the two branches of metabolism are utilized to different extents during development. TCA cycle genes are down-regulated while lipid metabolism genes are up-regulated during the dauer stage [49], and our analysis suggests that the two cytosolic superoxide dismutases have specialized to be active under specific metabolic conditions in worm. Interestingly the family of mitochondrial superoxide dismutases (SOD2, *sod-2, sod-3)* show a similar pattern of specialization, as can be examined through the web interface.

## Discussion

We have developed a method to leverage a large compendium of gene expression data to provide a measure of functional similarity of homologs across organisms. Our measure reliably predicts gene pairs that share tissue expression patterns or participate in the same biological process even for closely related genes and can therefore serve as a useful tool for identifying homologs with analogous function, as well as a way of examining more general questions about the landscape of functional similarity.

By leveraging a large compendium of expression data, our method yields both good gene coverage and extensive functional coverage by combining datasets from many tissues and perturbations. As expression datasets provide information for many genes that have not been studied in any other way, our method is, for many homologous gene-pairs, the only way currently available to explore functional relationships. Our method is also designed to allow detailed examination of the sources of our functional predictions through the provided web interface (available at http://networkhomologs.princeton.edu), maximizing its utility as an exploratory tool for biology researchers, especially in cases where functional similarity is complex and context dependent and no “best” ortholog exists.

## Methods

### Data and data processing

Microarray data for *S. cerevisiae* was identical to that used in [17] and *C. elegans* data was identical to that used in [50]. Raw CEL files for Data for *D. melanogaster* (DrosGenome1) and *M. musculus* (Mouse420_2) were downloaded from Gene Expression Omnibus (GEO) [51] and processed using Bioconductor [52] with VSN normalization [53] and probesets collapsed according to the algorithm described in [54]. See Text S1 for a list of datasets and sources.

### Bayesian integration of microarray data

Standards for Bayesian integration were constructed using a custom set of Gene Ontology terms described in [54]. A pair of genes is considered positive if both genes are experimentally annotated to the same specific GO term though homologous pairs were excluded from positive examples. Negative pairs were selected at random from the set of genes included in positive examples to give a prior probability of functional interaction of 0.05. Bayesian integration was performed as described in [17] using the BNCreator tool provided with our open-source Sleipnir library [55]. While there is a large number of publically available microarray experiment for *Mus musculus*, many datasets perform randomly with respect to this Gene Ontology standard and thus cannot meaningfully affect functional relationship probabilities. To avoid decreases in performance due to violations in the independence assumption and overfitting only the top 100 datasets (as measured by area under performance recall curve over the top 10% recall) were used in the integration of the mouse network (Small modificatin to this precedure did not affet the performance of the resulting network).

### Computing functional similarity across organism

Functional interaction networks were used to define gene neighborhoods. A “hard-cutoff” neighborhood of a query gene is defined as all genes connected to the query with a resonable probability. While the hard-cutoff of 0.5 is a natural probability cutoff (and was used by us in this paper), this too can be adjusted by the user in the on-line interface. As not all genes have a sufficient number of neighbors above this threshold, we have also set the minimal size of the neighborhood at 50, termed “soft-cutoff”. While using the soft-cutoff method slightly decreases our evaluation performance, we believe that it is nevertheless important to give scores for as many gene pairs as possible and we use this method throughout the paper. The soft-cutoff is the default option in our web interface though it can be turned off by the user. For the purpose of all evaluations and figures a hard-cutoff of 0.5 and a soft-cutoff of 50 is used. Based on our calculations a hard-cutoff of 0.5 performed best overall though may not be optimal for all queries.

After gene neighborhoods are defined based on our functional interaction networks, TreeFam B families [2] were used to map gene neighborhoods from different organisms onto a species-independent space of meta-genes. The TreeFam system defines families that evolved from a single gene in the last common ancestor of all animals (with closely related plant and fungi genes included). A small number of genes that appeared in more than one TreeFam family were excluded from consideration. To map a gene's neighborhood onto the meta-gene set, a meta-gene is considered present if any of the member genes are present, thus for multi-gene families a connection to any of the members is sufficient.

To determine the functional similarity of genes from different organisms, we compute the hypergeometric p-value of their meta-gene neighborhood overlap. The background set of TreeFam families used for the p-value computation is specific to the organism pair considered and is defined as all TreeFam families that contained at least one gene from each organism such that the gene is also present in our microarray compendium. Likewise for the purpose of the p-value calculation the size of each gene's TreeFam neighborhood is considered to be the set of those TreeFam families that are both present in the gene's neighborhood and in the organism-pair-specific background set.

### Evaluations

Our evaluation methodology is motivated by how we believe our system is likely to be used by biology researchers. In particular, given a query gene we would like to evaluate if our network similarity score produces a ranking of potential homologs that is consistent with what is known about the genes experimentally. We expect that homologs expressed in the same tissue or those that show the same phenotype as the query should be ranked above those that do not share these functional attributes. In order to evaluate this we define various standards for homolog pairings.

In the nervous system standard homolog pairs that both express in the nervous system are considered positive, while homolog pairs whose expression has been studied but were not co-expressed in the nervous system are considered negative. For Gene Ontology based evaluations we used a set of specific GO terms with experimental evidence codes (the same set that is used for standard construction). Homolog pairs that shared at least one such annotation were considered positive, while homolog pairs that have been experimentally annotated (to this set of specific GO terms) but did not have annotations in common are considered negatives (See Dataset S1 for all evaluation standards and annotation sources). To perform the evaluation we consider a single query gene with several homologs in another organism such that that at least one query-homolog pair would be considered positive and at least one would be considered negative according to a particular standard (nervous system standard or GO standard). Our evaluation is designed to determine if our functional similarity ranks the query-homolog pairs non-randomly relative to a particular standard. While it is possible to compute AUCs on a per-query basis, the set of query-homolog pairs defined in the standard is often quite small (for GO derived standards often there are only 2 pairs, the minimum possible). To increase the statistical power of this evaluation we combine results from all query genes by first normalizing their ranks so that the query-homolog pairs with the highest score receives a rank of 1 and the pair with lowest score receives the rank of 0 with the remaining pairs (if any) falling somewhere in between. The normalized ranks are then combined to compute a global AUC and determine significance.

### Treefam GO enrichments

To compute GO enrichments for Treefam families we consider a family to be annotated to a particular term if any of the member genes have an experimental annotation for that term. GO enrichment is computed as hypergeometric p-values with the background count taken from the organism-pairs-specific background families defined above. Thus, while the annotations are not organism-specific, the enrichment computation does depend on the organism pair being considered. All p-values are cutoff at and FDR of 0.05.

## Supporting Information

Dataset S1 Binary evaluation standards and annotation sources.
(4.51 MB TAR)

Figure S1 Average fraction of family wide experimental GO annotations that belong to the single most annotated family member. Experimental annotations may often show bias with respect to close homologs as some members of homologous families are studied and annotated more thoroughly than others.
(0.01 MB EPS)

Figure S2 Average fraction of family wide protein-protein interactions (as compiled by BioGRID) that belong to the single most connected family member. While closely related homologs would be expected to have similar numbers of interactions, due to various study biases the number of reported PPIs varies widely.
(0.01 MB EPS)

Table S1 The results of all evaluations performed, includes networks with added PPI informations and process-specific evaluations.
(0.03 MB XLS)

Text S1 A list of all datasets included in the integration and their sources.
(0.05 MB DOC)
