# Supplementary material for: Accurate Quantification of Functional Analogy among Close Homologs
Source: PLoS Comput Biol. 2011 Feb 3;7(2):e1001074. doi: 10.1371/journal.pcbi.1001074 (PMC3033368; doi:10.1371/journal.pcbi.1001074)
Supplement: Text S1 — A list of all datasets included in the integration and their sources. (0.05 MB DOC) [file pcbi.1001074.s005.doc]

Organism: *D. melanogaster*

platform: DrosGenome1

source: Gene Expression Ominbus (Raw CEL files)

GSE10012

GSE10013

GSE10014

GSE10053

GSE10264

GSE10781

GSE10853

GSE10940

GSE11046

GSE11047

GSE11164

GSE11203

GSE11231

GSE11825

GSE12332

GSE12477

GSE12834

GSE13328

GSE13723

GSE14058

GSE14531

GSE14756

GSE14780

GSE15092

GSE1690

GSE2422

GSE2623

GSE2780

GSE2828

GSE2863

GSE3057

GSE3060

GSE3069

GSE3072

GSE3566

GSE3826

GSE3828

GSE3829

GSE3830

GSE3831

GSE3832

GSE3842

GSE3854

GSE3990

GSE4032

GSE4174

GSE4188

GSE4235

GSE5321

GSE5404

GSE5430

GSE5984

GSE6141

GSE6300

GSE6418

GSE6420

GSE6490

GSE6491

GSE6492

GSE6493

GSE6515

GSE6542

GSE6558

GSE6655

GSE6714

GSE6928

GSE6994

GSE7110

GSE7159

GSE7614

GSE7655

GSE7763

GSE7772

GSE7873

GSE8330

GSE8619

GSE8620

GSE8623

GSE8722

GSE8751

GSE8775

GSE8892

GSE8938

GSE9001

GSE9088

GSE9107

GSE9149

GSE9271

GSE9425

GSE9538

GSE9552

GSE9571

GSE9889

Organism: *M. musculus*

platform: Mouse430_2

source: Gene Expression Ominbus (Raw CEL files)

GSE10246

GSE9954

GSE8249

GSE1986

GSE10634

GSE8836

GSE10871

GSE9249

GSE13044

GSE4189

GSE5671

GSE7798

GSE9892

GSE7460

GSE4818

GSE11382

GSE7342

GSE8316

GSE9725

GSE1479

GSE12581

GSE4098

GSE10744

GSE7020

GSE12421

GSE11201

GSE7897

GSE12609

GSE9013

GSE8292

GSE3653

GSE9441

GSE7324

GSE5657

GSE12075

GSE1871

GSE5333

GSE8128

GSE9355

GSE14415

GSE12248

GSE13948

GSE10290

GSE7683

GSE7357

GSE7676

GSE6998

GSE4288

GSE4230

GSE12982

GSE9809

GSE7685

GSE9763

GSE8790

GSE7793

GSE4307

GSE6980

GSE9465

GSE12430

GSE7475

GSE6689

GSE5976

GSE9566

GSE13692

GSE7657

GSE7219

GSE13981

GSE11818

GSE12078

GSE14891

GSE4308

GSE11662

GSE3889

GSE13231

GSE8024

GSE10392

GSE7688

GSE2372

GSE10525

GSE10813

GSE8512

GSE8396

GSE5817

GSE11165

GSE13409

GSE13149

GSE13379

GSE9760

GSE6933

GSE11973

GSE6259

GSE6055

GSE14431

GSE10765

GSE10493

GSE9444

GSE6957

GSE10964

GSE8039

GSE11703

Organism: *C. elegans*

platform: multiple

source: As used in [1], see “[http://worm-tissue.princeton.edu/search/bib_array](http://worm-tissue.princeton.edu/search)” for dataset description

Affy_develpment

Baugh_2003

Baugh_2005

Bishop_2004

Chen_2006

Colosimo_2004

Cui_2007

Custodia_2001

Denver_2005

Dinkova_2004

Dybbs_2005

Fox_2005

Fox_2007

Golden_2004

Golden_2008

GuhaThakurta_2002

Hill_2000

Jiang_2001

Kim_

Kniazeva_2004

Kwon_2004

Leacock_2006

Lee_2006

Li_2006

Link_2003

Liu_2004

Lund_2002

McCarroll_2004a

McCarroll_2004b

McElwee_2004

Menzel_2001

Murphy_2003

Nmbl

Pauli_2005

Portman_2004

Rajagopal_2008

Reichert_2005

Reinke_2000

Reinke_2004

Romagnolo_2002

Shapira_2006

Shen_2005

Small

Stetina_2007

Troemel_2006

Viswanathan_2005

Wang_2003

Wang_2006

Zhang_2007

Organism: *S. cerevisiae*

platform: multiple

source: As used in [2], see “http://imperio.princeton.edu:3000/yeast/dataset_listing” for dataset description

Bedalov01

Boer05

Brauer05_batch1

Brauer05_batch2

Brem02_set1

Brem02_set2

Bro03

Bulik03

Caba05

CarmelHarel01

Carroll01

Causton01_acid

Causton01_peroxide

Chitikila02

Cho98

DeRisi97

Duvel03_delayedRap

Epstein00

Fleming99

Fry03

Gasch00_carbonSources

Gasch00_diamideTreatment

Gasch00_DTT_y13_

Gasch00_DTT_y14_

Gasch00_HS25_37

Gasch00_HSmild

Gasch00_HSto37

Gasch00_hypo_osmotic

Gasch00_Ndepletion

Gasch00_stationaryPhase_y12_

Gasch00_stationaryPhase_y14_

Hardwick00

Hughes00

Ideker01

Jelinsky00

Jin04

Leber04

Lyons00

Martin04

ORourke03

Pitkanen04

Primig00

Roberts00

Sabet04

Saldanha04_LeucineBatchChem

Saldanha04_PhosphateBatchChem

Saldanha04_SulfateBatchChem

Saldanha04_UracilBatchChem

Saldanha04_UraSulPhoLeuComp

Sapra04

Schawalder04

Segal03_HSkin82

Segal03_stationaryPhaseYPL230W

Shapira04

Spellman98_alphaFactor

Spellman98_elutriation

Tai05

Takagi05

Williams02_subA

Williams02_subB

Williams02_subC

Williams02_subD

Yoshimoto02_Ca

Yoshimoto02_Na

Yvert03_Brem05

1. Chikina MD, Huttenhower C, Murphy CT, Troyanskaya OG (2009) Global prediction of tissue-specific gene expression and context-dependent gene networks in Caenorhabditis elegans. PLoS Comput Biol 5: e1000417.

2. Hibbs MA, Hess DC, Myers CL, Huttenhower C, Li K, et al. (2007) Exploring the functional landscape of gene expression: directed search of large microarray compendia. Bioinformatics 23: 2692-2699.
